# Methyl 4-benz­yloxy-7-meth­oxy-1-methyl-1*H*-indole-2-carboxyl­ate

**DOI:** 10.1107/S1600536812031236

**Published:** 2012-07-18

**Authors:** Peng Wang, Hualu Xing, Yang Liu, Wencheng Xie, Guisen Zhao

**Affiliations:** a School of Pharmaceutical Sciences, Shandong University, Jinan 250012, People’s Republic of China

## Abstract

There are two independent mol­ecules in the asymmetric unit of the title compound, C_19_H_19_NO_4_. The indole unit in each mol­ecule is essentially planar, with mean deviations of 0.017 (1) and 0.013 (1) Å and forms dihedral angles of 50.17 (7) and 26.05 (6)° with the phenyl ring. In the crystal, mol­ecules are linked by weak C–H⋯π inter­actions.

## Related literature

For the anti­tumor activity of substituted indole compounds, see: Ziedan *et al.* (2010 ▶). For the crystal structures of related compounds, see: Butcher *et al.* (2006 ▶, 2007 ▶); Harrison *et al.* (2006 ▶); Hu *et al.* (2005 ▶). For the synthesis of 5-benz­yloxy-7-bromo-1H-indole-2-carb­oxy­lic acid, see: Fresneda *et al.*(2001 ▶).
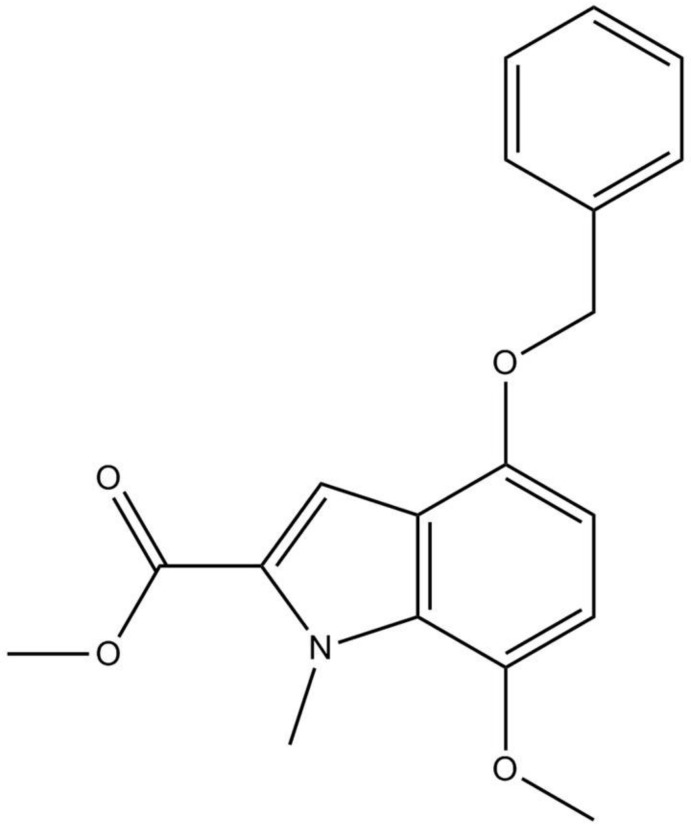


## Experimental

### 

#### Crystal data

C_19_H_19_NO_4_
*M* *_r_* = 325.35
Triclinic, 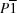

*a* = 7.622 (2) Å
*b* = 12.871 (4) Å
*c* = 16.928 (5) Å
α = 93.831 (3)°
β = 100.158 (3)°
γ = 93.456 (3)°
*V* = 1626.6 (8) Å^3^
*Z* = 4
Mo *K*α radiation
μ = 0.09 mm^−1^
*T* = 293 K
0.38 × 0.36 × 0.25 mm

#### Data collection

Bruker APEXII CCD area-detector diffractometer
Absorption correction: multi-scan (*SADABS*; Bruker, 2005 ▶) *T*_min_ = 0.965, *T*_max_ = 0.977
19234 measured reflections
7421 independent reflections
5286 reflections with *I* > 2σ(*I*)
*R*_int_ = 0.023

#### Refinement

*R*[*F* ^2^ > 2σ(*F* ^2^)] = 0.044
*wR*(*F* ^2^) = 0.134
*S* = 1.05
7421 reflections
440 parameters
H-atom parameters constrained
Δρ_max_ = 0.24 e Å^−3^
Δρ_min_ = −0.17 e Å^−3^

### 

Data collection: *APEX2* (Bruker, 2005 ▶); cell refinement: *SAINT* (Bruker, 2005 ▶); data reduction: *SAINT*; program(s) used to solve structure: *SHELXS97* (Sheldrick, 2008 ▶); program(s) used to refine structure: *SHELXL97* (Sheldrick, 2008 ▶); molecular graphics: *DIAMOND* (Brandenburg, 2008 ▶); software used to prepare material for publication: *PLATON* (Spek, 2009 ▶).

## Supplementary Material

Crystal structure: contains datablock(s) I, global. DOI: 10.1107/S1600536812031236/lx2234sup1.cif

Structure factors: contains datablock(s) I. DOI: 10.1107/S1600536812031236/lx2234Isup2.hkl

Supplementary material file. DOI: 10.1107/S1600536812031236/lx2234Isup3.cml

Additional supplementary materials:  crystallographic information; 3D view; checkCIF report

## Figures and Tables

**Table 1:** Hydrogen-bond geometry (Å, °) *Cg*1, *Cg*2, *Cg*3 and *Cg*4 are the centroids of the C20–C25 phenyl, C1–C6 phenyl, C27–C32 phenyl and C12–C15/N1 pyrrole rings, respectively.

*D*—H⋯*A*	*D*—H	H⋯*A*	*D*⋯*A*	*D*—H⋯*A*
C5—H5⋯*Cg*1	0.93	2.88	3.6778 (7)	145
C16—H16*A*⋯*Cg*2^i^	0.96	2.87	3.7812 (9)	158
C17—H17*C*⋯*Cg*3^ii^	0.96	2.90	3.845 (1)	167
C26—H26*A*⋯*Cg*4^iii^	0.96	2.94	3.7442 (8)	141

Symmetry codes: (i) 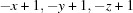; (ii) 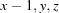; (iii) 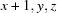.

**Fig. 1.**
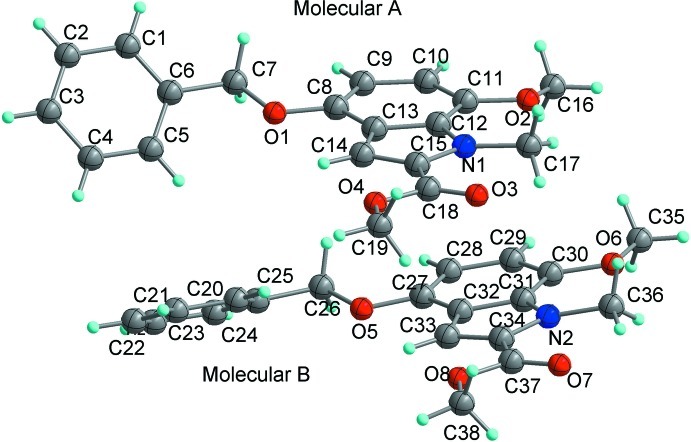
The molecular structure of the title compound with the atom numbering scheme. Displacement ellipsoids are drawn at the 50% probability level. H atoms are presented as small spheres of arbitrary radius.

**Fig. 2.**
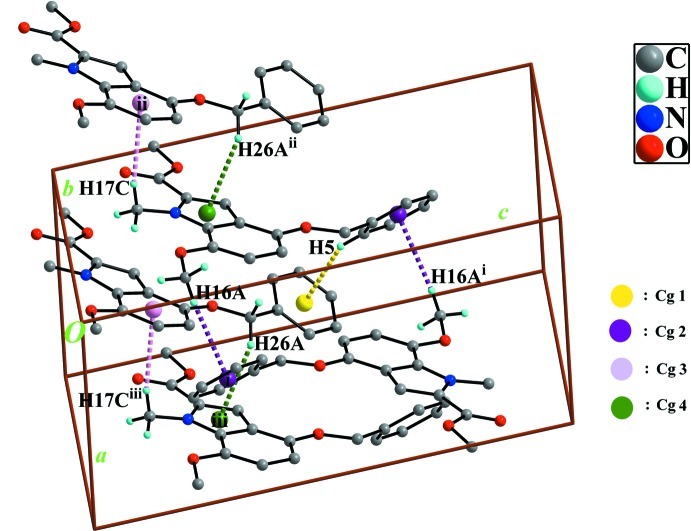
A view of the C–H···π interactions (dotted lines) in the crystal structure of the title compound. Cg denotes the ring centroids. [Symmetrycodes: (i) -x+1, -y+1, -z+1; (ii) x-1, y, z; (iii) x+1, y, z.]

## supplementary crystallographic information

### Comment

Substituted indole derivatives have attracted much attention due to their biological properties such as antitumor activities (Ziedan *et al.* 2010). Recently, the crystal structures of methyl 5-halo-1H-indole-2-carboxylate analogues were reported (Butcher *et al.*, 2006, 2007; Harrison *et al.*, 2006). We report herein the crystal structure of the title compound.

The asymmetric unit of the title compound is shown in Fig. 1. There are two independent unique molecules [labelled A & B] in which the indole unit is essentially planar, with mean deviations of 0.017 (1) Å for A and 0.013 (1) Å for B, respectively, from the least-squares plane defined by the nine constituent atoms. The crystal packing is stabilized by weak intermolecular C–H···π interactions (Table 1 & Fig. 2, Cg1, Cg2, Cg3 and Cg4 are the centroids of C20-C25 pheny ring, C1-C6 pheny ring, C27-C32 pheny ring and C12-C15/N1 pyrrol ring, respectively.

### Experimental

A mixture of 4-(benzyloxy)-7-bromo-1*H*-indole-2-carboxylic acid (Fresneda *et al.*, 2001) (0.35 g, 1 mmol), CuI (0.19 g, 1 mmol), CH_3_ONa (0.38 g, 7 mmol) in anhydrous CH_3_OH (2 ml) and DMF (4 ml) under N_2_ atmosphere was heated to reflux for 5 h. After cooling to r.t., the mixture was poured into water (50 ml) and acidified with aq. HCl (6 N) to pH 1-2. The precipitate was filtered, washed several times with water, dried under vacuum, and then dissolved in anhydrous DMF. NaH (0.04 g, 1.5 mmol) was added to the solution under 0 *^o^*C followed by dimethyl sulfate (0.19 g, 1.5 mmol). The mixture was stired at r.t. for 4 h, and then was poured into ice-cold water. The precipitate was filtered, washed several times with water, and further purified by column chromatography (10% EtOAc/ Petroleum ether) and recrystallization from 10% EtOAc/Petroleum ether gave 0.21 g (64%) of white crystals. Crystals of X-ray diffraction quality were obtained by recrystallization from CH_2_Cl_2_/n-hexane mixture (4:1).

### Refinement

All H atoms were placed geometrically and treated as riding on their parent atoms with C–H = 0.96 Å (methyl) or 0.93 Å (aromatic and methenyl), 0.82 Å (hydroxyl) and *U*_iso_(H) = 1.2*U*_eq_(C or O).

### Crystal data

**Table:**

C_19_H_19_NO_4_	*Z* = 4
*M**_r_* = 325.35	*F*(000) = 688
Triclinic, *P*1	*D*_x_ = 1.329 Mg m^−^^3^
Hall symbol: -P 1	Melting point = 373.1–374.8 K
*a* = 7.622 (2) Å	Mo *K*α radiation, λ = 0.71069 Å
*b* = 12.871 (4) Å	Cell parameters from 6012 reflections
*c* = 16.928 (5) Å	θ = 2.5–27.2°
α = 93.831 (3)°	µ = 0.09 mm^−^^1^
β = 100.158 (3)°	*T* = 293 K
γ = 93.456 (3)°	Block, colourless
*V* = 1626.6 (8) Å^3^	0.38 × 0.36 × 0.25 mm

### Data collection

**Table:**

Bruker APEXII CCD area-detector diffractometer	7421 independent reflections
Radiation source: fine-focus sealed tube	5286 reflections with *I* > 2σ(*I*)
Graphite monochromator	*R*_int_ = 0.023
φ and ω scans	θ_max_ = 27.7°, θ_min_ = 1.2°
Absorption correction: multi-scan (*SADABS*; Bruker, 2005)	*h* = −9→9
*T*_min_ = 0.965, *T*_max_ = 0.977	*k* = −16→16
19234 measured reflections	*l* = −21→22

### Refinement

**Table:**

Refinement on *F*^2^	Secondary atom site location: difference Fourier map
Least-squares matrix: full	Hydrogen site location: inferred from neighbouring sites
*R*[*F*^2^ > 2σ(*F*^2^)] = 0.044	H-atom parameters constrained
*wR*(*F*^2^) = 0.134	*w* = 1/[σ^2^(*F*_o_^2^) + (0.0655*P*)^2^ + 0.2116*P*] where *P* = (*F*_o_^2^ + 2*F*_c_^2^)/3
*S* = 1.05	(Δ/σ)_max_ < 0.001
7421 reflections	Δρ_max_ = 0.24 e Å^−^^3^
440 parameters	Δρ_min_ = −0.17 e Å^−^^3^
0 restraints	Extinction correction: *SHELXL97* (Sheldrick, 2008), Fc^*^=kFc[1+0.001xFc^2^λ^3^/sin(2θ)]^-1/4^
Primary atom site location: structure-invariant direct methods	Extinction coefficient: 0.0035 (9)

### Special details

**Table:**

Geometry. All esds (except the esd in the dihedral angle between two l.s. planes) are estimated using the full covariance matrix. The cell esds are taken into account individually in the estimation of esds in distances, angles and torsion angles; correlations between esds in cell parameters are only used when they are defined by crystal symmetry. An approximate (isotropic) treatment of cell esds is used for estimating esds involving l.s. planes.
Refinement. Refinement of F^2^ against ALL reflections. The weighted R-factor wR and goodness of fit S are based on F^2^, conventional R-factors R are based on F, with F set to zero for negative F^2^. The threshold expression of F^2^ > 2sigma(F^2^) is used only for calculating R-factors(gt) etc. and is not relevant to the choice of reflections for refinement. R-factors based on F^2^ are statistically about twice as large as those based on F, and R- factors based on ALL data will be even larger.

### Fractional atomic coordinates and isotropic or equivalent isotropic displacement parameters (Å^2^)

**Table:**

	*x*	*y*	*z*	*U*_iso_*/*U*_eq_	
C1	0.3201 (2)	0.71878 (13)	0.71353 (9)	0.0534 (4)	
H1	0.2474	0.6626	0.7240	0.064*	
C2	0.3716 (3)	0.80075 (15)	0.77138 (10)	0.0667 (5)	
H2	0.3344	0.7992	0.8208	0.080*	
C3	0.4770 (3)	0.88439 (14)	0.75641 (11)	0.0694 (5)	
H3	0.5113	0.9396	0.7955	0.083*	
C4	0.5321 (3)	0.88654 (14)	0.68313 (11)	0.0650 (5)	
H4	0.6029	0.9436	0.6727	0.078*	
C5	0.4826 (2)	0.80460 (12)	0.62552 (9)	0.0544 (4)	
H5	0.5210	0.8063	0.5764	0.065*	
C6	0.37566 (19)	0.71944 (11)	0.64011 (8)	0.0443 (3)	
C7	0.3246 (2)	0.62797 (12)	0.57930 (8)	0.0493 (4)	
H7A	0.4215	0.5820	0.5825	0.059*	
H7B	0.2198	0.5888	0.5901	0.059*	
C8	0.23533 (19)	0.59066 (11)	0.43789 (8)	0.0437 (3)	
C9	0.2426 (2)	0.48538 (12)	0.44087 (9)	0.0511 (4)	
H9	0.2843	0.4582	0.4897	0.061*	
C10	0.1877 (2)	0.41722 (11)	0.37112 (9)	0.0505 (4)	
H10	0.1968	0.3459	0.3749	0.061*	
C11	0.12125 (19)	0.45221 (10)	0.29780 (8)	0.0423 (3)	
C12	0.10795 (17)	0.56092 (10)	0.29461 (8)	0.0385 (3)	
C13	0.16855 (18)	0.63004 (10)	0.36340 (8)	0.0396 (3)	
C14	0.14128 (19)	0.73230 (11)	0.34046 (8)	0.0434 (3)	
H14	0.1698	0.7939	0.3734	0.052*	
C15	0.06500 (19)	0.72338 (11)	0.26068 (8)	0.0427 (3)	
C16	0.1099 (3)	0.28331 (12)	0.23069 (10)	0.0602 (4)	
H16A	0.2371	0.2812	0.2450	0.090*	
H16B	0.0687	0.2469	0.1787	0.090*	
H16C	0.0536	0.2505	0.2700	0.090*	
C17	−0.0428 (2)	0.57773 (12)	0.15155 (8)	0.0530 (4)	
H17A	−0.0089	0.5081	0.1415	0.079*	
H17B	−0.0071	0.6212	0.1124	0.079*	
H17C	−0.1700	0.5764	0.1478	0.079*	
C18	0.0067 (2)	0.80779 (12)	0.21026 (9)	0.0494 (4)	
C19	−0.0144 (4)	0.98951 (14)	0.21080 (13)	0.0948 (8)	
H19A	0.0385	0.9929	0.1635	0.142*	
H19B	0.0219	1.0519	0.2456	0.142*	
H19C	−0.1423	0.9833	0.1955	0.142*	
C20	0.8907 (2)	0.78348 (12)	0.49954 (9)	0.0503 (4)	
H20	0.9494	0.7222	0.5012	0.060*	
C21	0.9458 (2)	0.86294 (13)	0.55865 (9)	0.0571 (4)	
H21	1.0398	0.8545	0.6004	0.069*	
C22	0.8629 (2)	0.95491 (14)	0.55648 (10)	0.0606 (4)	
H22	0.9003	1.0087	0.5965	0.073*	
C23	0.7236 (2)	0.96653 (14)	0.49423 (11)	0.0623 (4)	
H23	0.6681	1.0289	0.4919	0.075*	
C24	0.6664 (2)	0.88617 (13)	0.43554 (10)	0.0567 (4)	
H24	0.5714	0.8945	0.3943	0.068*	
C25	0.74889 (19)	0.79327 (12)	0.43736 (8)	0.0463 (3)	
C26	0.6865 (2)	0.70257 (12)	0.37704 (9)	0.0519 (4)	
H26A	0.7848	0.6594	0.3727	0.062*	
H26B	0.5928	0.6604	0.3944	0.062*	
C27	0.5479 (2)	0.66508 (12)	0.24058 (8)	0.0466 (3)	
C28	0.5498 (2)	0.55956 (12)	0.24361 (9)	0.0527 (4)	
H28	0.6048	0.5323	0.2905	0.063*	
C29	0.4701 (2)	0.49140 (12)	0.17701 (9)	0.0529 (4)	
H29	0.4747	0.4199	0.1811	0.064*	
C30	0.3858 (2)	0.52585 (11)	0.10641 (9)	0.0478 (3)	
C31	0.38111 (19)	0.63503 (11)	0.10291 (8)	0.0432 (3)	
C32	0.46230 (19)	0.70394 (11)	0.16862 (8)	0.0438 (3)	
C33	0.4334 (2)	0.80607 (12)	0.14658 (9)	0.0482 (3)	
H33	0.4731	0.8677	0.1782	0.058*	
C34	0.3362 (2)	0.79755 (11)	0.06999 (8)	0.0466 (3)	
C35	0.3145 (3)	0.35453 (13)	0.04259 (11)	0.0668 (5)	
H35A	0.4376	0.3388	0.0539	0.100*	
H35B	0.2579	0.3197	−0.0082	0.100*	
H35C	0.2549	0.3311	0.0843	0.100*	
C36	0.1910 (3)	0.65126 (14)	−0.03299 (10)	0.0663 (5)	
H36A	0.2483	0.5958	−0.0566	0.099*	
H36B	0.1735	0.7054	−0.0693	0.099*	
H36C	0.0774	0.6248	−0.0227	0.099*	
C37	0.2737 (2)	0.88262 (12)	0.02112 (9)	0.0518 (4)	
C38	0.2686 (3)	1.06634 (14)	0.02114 (13)	0.0808 (6)	
H38A	0.3072	1.0631	−0.0299	0.121*	
H38B	0.3242	1.1278	0.0533	0.121*	
H38C	0.1411	1.0689	0.0128	0.121*	
N1	0.04466 (15)	0.61930 (9)	0.23182 (6)	0.0408 (3)	
N2	0.30332 (16)	0.69353 (9)	0.04256 (7)	0.0461 (3)	
O1	0.28763 (15)	0.66482 (8)	0.50106 (6)	0.0538 (3)	
O2	0.06565 (15)	0.38876 (7)	0.22827 (6)	0.0517 (3)	
O3	−0.06518 (19)	0.79859 (9)	0.14086 (7)	0.0740 (4)	
O4	0.0436 (2)	0.90008 (8)	0.25245 (7)	0.0729 (4)	
O5	0.62046 (16)	0.73933 (8)	0.30083 (6)	0.0570 (3)	
O6	0.30467 (18)	0.46313 (9)	0.03939 (7)	0.0664 (3)	
O7	0.1933 (2)	0.87380 (10)	−0.04683 (7)	0.0833 (4)	
O8	0.31871 (19)	0.97476 (9)	0.06225 (7)	0.0706 (4)	

### Atomic displacement parameters (Å^2^)

**Table:**

	*U*^11^	*U*^22^	*U*^33^	*U*^12^	*U*^13^	*U*^23^
C1	0.0610 (9)	0.0577 (9)	0.0429 (8)	0.0109 (7)	0.0091 (7)	0.0090 (7)
C2	0.0911 (13)	0.0698 (12)	0.0413 (9)	0.0209 (10)	0.0138 (8)	0.0030 (8)
C3	0.0982 (14)	0.0530 (10)	0.0510 (10)	0.0162 (10)	−0.0025 (9)	−0.0069 (8)
C4	0.0822 (12)	0.0496 (9)	0.0588 (10)	0.0031 (8)	−0.0005 (9)	0.0087 (8)
C5	0.0671 (10)	0.0535 (9)	0.0430 (8)	0.0084 (8)	0.0074 (7)	0.0098 (7)
C6	0.0488 (8)	0.0478 (8)	0.0353 (7)	0.0145 (6)	−0.0011 (6)	0.0094 (6)
C7	0.0604 (9)	0.0511 (9)	0.0346 (7)	0.0122 (7)	−0.0008 (6)	0.0093 (6)
C8	0.0506 (8)	0.0450 (8)	0.0334 (7)	0.0081 (6)	0.0000 (6)	0.0049 (6)
C9	0.0663 (10)	0.0472 (8)	0.0381 (7)	0.0113 (7)	−0.0010 (7)	0.0117 (6)
C10	0.0667 (10)	0.0371 (7)	0.0470 (8)	0.0084 (7)	0.0042 (7)	0.0098 (6)
C11	0.0480 (8)	0.0365 (7)	0.0406 (7)	0.0024 (6)	0.0039 (6)	0.0033 (6)
C12	0.0411 (7)	0.0389 (7)	0.0347 (7)	0.0050 (5)	0.0027 (5)	0.0065 (5)
C13	0.0447 (7)	0.0389 (7)	0.0340 (7)	0.0048 (6)	0.0021 (5)	0.0054 (5)
C14	0.0553 (8)	0.0374 (7)	0.0352 (7)	0.0057 (6)	0.0006 (6)	0.0032 (6)
C15	0.0516 (8)	0.0382 (7)	0.0365 (7)	0.0050 (6)	0.0017 (6)	0.0048 (6)
C16	0.0812 (12)	0.0400 (8)	0.0578 (10)	0.0107 (8)	0.0073 (8)	0.0002 (7)
C17	0.0698 (10)	0.0472 (9)	0.0358 (7)	0.0045 (7)	−0.0061 (7)	0.0002 (6)
C18	0.0644 (10)	0.0424 (8)	0.0387 (8)	0.0060 (7)	−0.0004 (7)	0.0074 (6)
C19	0.158 (2)	0.0454 (10)	0.0725 (13)	0.0247 (12)	−0.0136 (13)	0.0174 (9)
C20	0.0536 (9)	0.0557 (9)	0.0411 (8)	0.0099 (7)	0.0023 (6)	0.0125 (7)
C21	0.0607 (10)	0.0669 (11)	0.0409 (8)	0.0036 (8)	−0.0011 (7)	0.0114 (7)
C22	0.0721 (11)	0.0587 (10)	0.0494 (9)	−0.0009 (8)	0.0093 (8)	0.0030 (8)
C23	0.0698 (11)	0.0544 (10)	0.0645 (11)	0.0141 (8)	0.0117 (9)	0.0097 (8)
C24	0.0536 (9)	0.0617 (10)	0.0535 (9)	0.0142 (8)	−0.0007 (7)	0.0139 (8)
C25	0.0485 (8)	0.0547 (9)	0.0367 (7)	0.0058 (7)	0.0060 (6)	0.0125 (6)
C26	0.0571 (9)	0.0560 (9)	0.0404 (8)	0.0066 (7)	−0.0019 (6)	0.0146 (7)
C27	0.0513 (8)	0.0490 (8)	0.0373 (7)	0.0040 (6)	0.0008 (6)	0.0070 (6)
C28	0.0612 (9)	0.0512 (9)	0.0439 (8)	0.0076 (7)	−0.0005 (7)	0.0148 (7)
C29	0.0634 (10)	0.0421 (8)	0.0527 (9)	0.0066 (7)	0.0050 (7)	0.0108 (7)
C30	0.0539 (9)	0.0416 (8)	0.0455 (8)	0.0029 (6)	0.0030 (6)	0.0040 (6)
C31	0.0466 (8)	0.0441 (8)	0.0381 (7)	0.0040 (6)	0.0039 (6)	0.0077 (6)
C32	0.0481 (8)	0.0437 (8)	0.0386 (7)	0.0039 (6)	0.0032 (6)	0.0072 (6)
C33	0.0597 (9)	0.0419 (8)	0.0397 (7)	0.0034 (7)	−0.0011 (6)	0.0051 (6)
C34	0.0563 (9)	0.0429 (8)	0.0387 (7)	0.0060 (6)	0.0013 (6)	0.0058 (6)
C35	0.0804 (12)	0.0481 (9)	0.0675 (11)	0.0063 (8)	0.0030 (9)	0.0002 (8)
C36	0.0839 (12)	0.0567 (10)	0.0470 (9)	−0.0044 (9)	−0.0161 (8)	0.0048 (8)
C37	0.0668 (10)	0.0470 (9)	0.0395 (8)	0.0093 (7)	0.0008 (7)	0.0062 (6)
C38	0.1103 (16)	0.0470 (10)	0.0798 (13)	0.0149 (10)	−0.0062 (12)	0.0201 (9)
N1	0.0491 (7)	0.0377 (6)	0.0332 (6)	0.0037 (5)	0.0001 (5)	0.0040 (5)
N2	0.0556 (7)	0.0432 (7)	0.0363 (6)	0.0041 (5)	−0.0015 (5)	0.0048 (5)
O1	0.0787 (7)	0.0457 (6)	0.0319 (5)	0.0086 (5)	−0.0060 (5)	0.0053 (4)
O2	0.0713 (7)	0.0362 (5)	0.0436 (6)	0.0049 (5)	−0.0003 (5)	0.0008 (4)
O3	0.1119 (10)	0.0530 (7)	0.0456 (6)	0.0096 (7)	−0.0206 (6)	0.0104 (5)
O4	0.1233 (11)	0.0381 (6)	0.0489 (6)	0.0168 (6)	−0.0125 (7)	0.0074 (5)
O5	0.0752 (7)	0.0518 (6)	0.0374 (5)	0.0025 (5)	−0.0093 (5)	0.0096 (5)
O6	0.0916 (9)	0.0459 (6)	0.0536 (7)	0.0038 (6)	−0.0079 (6)	0.0012 (5)
O7	0.1312 (12)	0.0600 (8)	0.0467 (7)	0.0181 (8)	−0.0221 (7)	0.0085 (6)
O8	0.1054 (10)	0.0435 (6)	0.0539 (7)	0.0109 (6)	−0.0138 (6)	0.0084 (5)

### Geometric parameters (Å, º)

**Table:**

C1—C2	1.379 (2)	C20—C21	1.374 (2)
C1—C6	1.382 (2)	C20—C25	1.388 (2)
C1—H1	0.9300	C20—H20	0.9300
C2—C3	1.368 (3)	C21—C22	1.375 (2)
C2—H2	0.9300	C21—H21	0.9300
C3—C4	1.380 (3)	C22—C23	1.380 (2)
C3—H3	0.9300	C22—H22	0.9300
C4—C5	1.375 (2)	C23—C24	1.379 (2)
C4—H4	0.9300	C23—H23	0.9300
C5—C6	1.388 (2)	C24—C25	1.384 (2)
C5—H5	0.9300	C24—H24	0.9300
C6—C7	1.496 (2)	C25—C26	1.495 (2)
C7—O1	1.4236 (16)	C26—O5	1.4222 (17)
C7—H7A	0.9700	C26—H26A	0.9700
C7—H7B	0.9700	C26—H26B	0.9700
C8—C9	1.363 (2)	C27—C28	1.363 (2)
C8—O1	1.3715 (17)	C27—O5	1.3661 (17)
C8—C13	1.4129 (18)	C27—C32	1.4144 (19)
C9—C10	1.408 (2)	C28—C29	1.404 (2)
C9—H9	0.9300	C28—H28	0.9300
C10—C11	1.371 (2)	C29—C30	1.368 (2)
C10—H10	0.9300	C29—H29	0.9300
C11—O2	1.3732 (16)	C30—O6	1.3752 (18)
C11—C12	1.4129 (19)	C30—C31	1.413 (2)
C12—N1	1.3772 (16)	C31—N2	1.3802 (18)
C12—C13	1.4082 (18)	C31—C32	1.4022 (19)
C13—C14	1.4139 (19)	C32—C33	1.410 (2)
C14—C15	1.3672 (19)	C33—C34	1.369 (2)
C14—H14	0.9300	C33—H33	0.9300
C15—N1	1.3850 (17)	C34—N2	1.3807 (18)
C15—C18	1.4679 (19)	C34—C37	1.468 (2)
C16—O2	1.4196 (18)	C35—O6	1.409 (2)
C16—H16A	0.9600	C35—H35A	0.9600
C16—H16B	0.9600	C35—H35B	0.9600
C16—H16C	0.9600	C35—H35C	0.9600
C17—N1	1.4551 (17)	C36—N2	1.4591 (19)
C17—H17A	0.9600	C36—H36A	0.9600
C17—H17B	0.9600	C36—H36B	0.9600
C17—H17C	0.9600	C36—H36C	0.9600
C18—O3	1.2005 (18)	C37—O7	1.1986 (18)
C18—O4	1.3325 (18)	C37—O8	1.3287 (18)
C19—O4	1.441 (2)	C38—O8	1.446 (2)
C19—H19A	0.9600	C38—H38A	0.9600
C19—H19B	0.9600	C38—H38B	0.9600
C19—H19C	0.9600	C38—H38C	0.9600
			
C2—C1—C6	120.56 (16)	C21—C22—C23	119.26 (16)
C2—C1—H1	119.7	C21—C22—H22	120.4
C6—C1—H1	119.7	C23—C22—H22	120.4
C3—C2—C1	120.31 (16)	C24—C23—C22	120.38 (16)
C3—C2—H2	119.8	C24—C23—H23	119.8
C1—C2—H2	119.8	C22—C23—H23	119.8
C2—C3—C4	119.77 (17)	C23—C24—C25	120.72 (15)
C2—C3—H3	120.1	C23—C24—H24	119.6
C4—C3—H3	120.1	C25—C24—H24	119.6
C5—C4—C3	120.18 (17)	C24—C25—C20	118.19 (14)
C5—C4—H4	119.9	C24—C25—C26	122.53 (13)
C3—C4—H4	119.9	C20—C25—C26	119.24 (14)
C4—C5—C6	120.49 (15)	O5—C26—C25	109.67 (12)
C4—C5—H5	119.8	O5—C26—H26A	109.7
C6—C5—H5	119.8	C25—C26—H26A	109.7
C1—C6—C5	118.69 (14)	O5—C26—H26B	109.7
C1—C6—C7	119.86 (14)	C25—C26—H26B	109.7
C5—C6—C7	121.42 (13)	H26A—C26—H26B	108.2
O1—C7—C6	108.96 (12)	C28—C27—O5	126.64 (13)
O1—C7—H7A	109.9	C28—C27—C32	118.10 (13)
C6—C7—H7A	109.9	O5—C27—C32	115.26 (13)
O1—C7—H7B	109.9	C27—C28—C29	120.96 (14)
C6—C7—H7B	109.9	C27—C28—H28	119.5
H7A—C7—H7B	108.3	C29—C28—H28	119.5
C9—C8—O1	126.36 (13)	C30—C29—C28	122.72 (14)
C9—C8—C13	118.56 (13)	C30—C29—H29	118.6
O1—C8—C13	115.09 (12)	C28—C29—H29	118.6
C8—C9—C10	120.92 (13)	C29—C30—O6	125.43 (14)
C8—C9—H9	119.5	C29—C30—C31	116.82 (13)
C10—C9—H9	119.5	O6—C30—C31	117.75 (13)
C11—C10—C9	122.34 (13)	N2—C31—C32	108.04 (12)
C11—C10—H10	118.8	N2—C31—C30	130.95 (13)
C9—C10—H10	118.8	C32—C31—C30	121.00 (13)
C10—C11—O2	124.41 (13)	C31—C32—C33	107.24 (12)
C10—C11—C12	117.17 (13)	C31—C32—C27	120.38 (13)
O2—C11—C12	118.42 (12)	C33—C32—C27	132.35 (13)
N1—C12—C13	107.93 (11)	C34—C33—C32	107.24 (13)
N1—C12—C11	131.19 (12)	C34—C33—H33	126.4
C13—C12—C11	120.88 (12)	C32—C33—H33	126.4
C12—C13—C8	120.06 (12)	C33—C34—N2	109.64 (12)
C12—C13—C14	107.30 (11)	C33—C34—C37	127.48 (14)
C8—C13—C14	132.60 (13)	N2—C34—C37	122.87 (13)
C15—C14—C13	107.01 (12)	O6—C35—H35A	109.5
C15—C14—H14	126.5	O6—C35—H35B	109.5
C13—C14—H14	126.5	H35A—C35—H35B	109.5
C14—C15—N1	109.88 (12)	O6—C35—H35C	109.5
C14—C15—C18	127.42 (13)	H35A—C35—H35C	109.5
N1—C15—C18	122.69 (12)	H35B—C35—H35C	109.5
O2—C16—H16A	109.5	N2—C36—H36A	109.5
O2—C16—H16B	109.5	N2—C36—H36B	109.5
H16A—C16—H16B	109.5	H36A—C36—H36B	109.5
O2—C16—H16C	109.5	N2—C36—H36C	109.5
H16A—C16—H16C	109.5	H36A—C36—H36C	109.5
H16B—C16—H16C	109.5	H36B—C36—H36C	109.5
N1—C17—H17A	109.5	O7—C37—O8	122.61 (14)
N1—C17—H17B	109.5	O7—C37—C34	126.56 (15)
H17A—C17—H17B	109.5	O8—C37—C34	110.83 (13)
N1—C17—H17C	109.5	O8—C38—H38A	109.5
H17A—C17—H17C	109.5	O8—C38—H38B	109.5
H17B—C17—H17C	109.5	H38A—C38—H38B	109.5
O3—C18—O4	122.79 (14)	O8—C38—H38C	109.5
O3—C18—C15	126.74 (14)	H38A—C38—H38C	109.5
O4—C18—C15	110.46 (12)	H38B—C38—H38C	109.5
O4—C19—H19A	109.5	C12—N1—C15	107.88 (11)
O4—C19—H19B	109.5	C12—N1—C17	125.66 (12)
H19A—C19—H19B	109.5	C15—N1—C17	126.24 (11)
O4—C19—H19C	109.5	C31—N2—C34	107.83 (11)
H19A—C19—H19C	109.5	C31—N2—C36	125.27 (13)
H19B—C19—H19C	109.5	C34—N2—C36	126.61 (13)
C21—C20—C25	121.04 (15)	C8—O1—C7	116.60 (11)
C21—C20—H20	119.5	C11—O2—C16	116.44 (11)
C25—C20—H20	119.5	C18—O4—C19	116.04 (13)
C20—C21—C22	120.38 (15)	C27—O5—C26	116.35 (12)
C20—C21—H21	119.8	C30—O6—C35	117.35 (13)
C22—C21—H21	119.8	C37—O8—C38	117.08 (13)
			
C6—C1—C2—C3	0.7 (3)	C29—C30—C31—N2	−177.77 (15)
C1—C2—C3—C4	−0.1 (3)	O6—C30—C31—N2	1.7 (2)
C2—C3—C4—C5	−0.5 (3)	C29—C30—C31—C32	1.1 (2)
C3—C4—C5—C6	0.5 (3)	O6—C30—C31—C32	−179.44 (13)
C2—C1—C6—C5	−0.6 (2)	N2—C31—C32—C33	−0.50 (16)
C2—C1—C6—C7	177.57 (14)	C30—C31—C32—C33	−179.63 (14)
C4—C5—C6—C1	0.0 (2)	N2—C31—C32—C27	177.89 (13)
C4—C5—C6—C7	−178.14 (14)	C30—C31—C32—C27	−1.2 (2)
C1—C6—C7—O1	142.32 (14)	C28—C27—C32—C31	0.4 (2)
C5—C6—C7—O1	−39.53 (19)	O5—C27—C32—C31	−179.26 (13)
O1—C8—C9—C10	−178.78 (14)	C28—C27—C32—C33	178.38 (16)
C13—C8—C9—C10	1.1 (2)	O5—C27—C32—C33	−1.3 (2)
C8—C9—C10—C11	−1.4 (2)	C31—C32—C33—C34	0.52 (17)
C9—C10—C11—O2	−179.80 (14)	C27—C32—C33—C34	−177.60 (16)
C9—C10—C11—C12	−0.6 (2)	C32—C33—C34—N2	−0.35 (18)
C10—C11—C12—N1	−178.50 (14)	C32—C33—C34—C37	−179.86 (15)
O2—C11—C12—N1	0.8 (2)	C33—C34—C37—O7	177.51 (18)
C10—C11—C12—C13	2.9 (2)	N2—C34—C37—O7	−1.9 (3)
O2—C11—C12—C13	−177.83 (12)	C33—C34—C37—O8	−2.2 (2)
N1—C12—C13—C8	177.81 (13)	N2—C34—C37—O8	178.31 (14)
C11—C12—C13—C8	−3.3 (2)	C13—C12—N1—C15	−0.05 (15)
N1—C12—C13—C14	−0.37 (15)	C11—C12—N1—C15	−178.80 (14)
C11—C12—C13—C14	178.54 (13)	C13—C12—N1—C17	−174.95 (13)
C9—C8—C13—C12	1.2 (2)	C11—C12—N1—C17	6.3 (2)
O1—C8—C13—C12	−178.89 (12)	C14—C15—N1—C12	0.47 (16)
C9—C8—C13—C14	178.88 (15)	C18—C15—N1—C12	−178.30 (14)
O1—C8—C13—C14	−1.3 (2)	C14—C15—N1—C17	175.33 (14)
C12—C13—C14—C15	0.64 (16)	C18—C15—N1—C17	−3.4 (2)
C8—C13—C14—C15	−177.22 (16)	C32—C31—N2—C34	0.29 (16)
C13—C14—C15—N1	−0.69 (17)	C30—C31—N2—C34	179.30 (15)
C13—C14—C15—C18	178.00 (15)	C32—C31—N2—C36	−173.88 (14)
C14—C15—C18—O3	−177.52 (17)	C30—C31—N2—C36	5.1 (3)
N1—C15—C18—O3	1.0 (3)	C33—C34—N2—C31	0.04 (17)
C14—C15—C18—O4	2.1 (2)	C37—C34—N2—C31	179.58 (14)
N1—C15—C18—O4	−179.32 (13)	C33—C34—N2—C36	174.12 (15)
C25—C20—C21—C22	−1.1 (2)	C37—C34—N2—C36	−6.3 (2)
C20—C21—C22—C23	0.0 (3)	C9—C8—O1—C7	−11.2 (2)
C21—C22—C23—C24	1.0 (3)	C13—C8—O1—C7	168.92 (13)
C22—C23—C24—C25	−0.9 (3)	C6—C7—O1—C8	−178.88 (12)
C23—C24—C25—C20	−0.2 (2)	C10—C11—O2—C16	−11.5 (2)
C23—C24—C25—C26	177.36 (15)	C12—C11—O2—C16	169.30 (13)
C21—C20—C25—C24	1.2 (2)	O3—C18—O4—C19	2.4 (3)
C21—C20—C25—C26	−176.43 (14)	C15—C18—O4—C19	−177.29 (18)
C24—C25—C26—O5	34.3 (2)	C28—C27—O5—C26	−7.6 (2)
C20—C25—C26—O5	−148.11 (14)	C32—C27—O5—C26	172.04 (13)
O5—C27—C28—C29	−179.94 (14)	C25—C26—O5—C27	−175.97 (12)
C32—C27—C28—C29	0.4 (2)	C29—C30—O6—C35	−2.4 (2)
C27—C28—C29—C30	−0.5 (3)	C31—C30—O6—C35	178.20 (15)
C28—C29—C30—O6	−179.67 (15)	O7—C37—O8—C38	−0.8 (3)
C28—C29—C30—C31	−0.3 (2)	C34—C37—O8—C38	178.94 (17)

### Hydrogen-bond geometry (Å, º)

Cg1, Cg2, Cg3 and Cg4 are the centroids of the C20–C25 phenyl ring, C1–C6 phenyl ring, C27–C32 phenyl ring and C12–C15/N1 pyrrole ring, respectively.

**Table:**

*D*—H···*A*	*D*—H	H···*A*	*D*···*A*	*D*—H···*A*
C5—H5···*Cg*1	0.93	2.88	3.6778 (7)	145
C16—H16*A*···*Cg*2^i^	0.96	2.87	3.7812 (9)	158
C17—H17*C*···*Cg*3^ii^	0.96	2.90	3.845 (1)	167
C26—H26*A*···*Cg*4^iii^	0.96	2.94	3.7442 (8)	141

Symmetry codes: (i) −*x*+1, −*y*+1, −*z*+1; (ii) *x*−1, *y*, *z*; (iii) *x*+1, *y*, *z*.
